# Cancer-ID: Toward Identification of Cancer by Tumor-Derived Extracellular Vesicles in Blood

**DOI:** 10.3389/fonc.2020.00608

**Published:** 2020-06-04

**Authors:** L. G. Rikkert, P. Beekman, J. Caro, F. A. W. Coumans, A. Enciso-Martinez, G. Jenster, S. Le Gac, W. Lee, T. G. van Leeuwen, G. B. Loozen, A. Nanou, R. Nieuwland, H. L. Offerhaus, C. Otto, D. M. Pegtel, M. C. Piontek, E. van der Pol, L. de Rond, W. H. Roos, R. B. M. Schasfoort, M. H. M. Wauben, H. Zuilhof, L. W. M. M. Terstappen

**Affiliations:** ^1^Department of Medical Cell Biophysics, University of Twente, Enschede, Netherlands; ^2^Laboratory of Experimental Clinical Chemistry, Amsterdam UMC, University of Amsterdam, Amsterdam, Netherlands; ^3^Vesicle Observation Center, Amsterdam UMC, University of Amsterdam, Amsterdam, Netherlands; ^4^Laboratory of Organic Chemistry, Wageningen University, Wageningen, Netherlands; ^5^Applied Microfluidics for Bioengineering Research, University of Twente, Enschede, Netherlands; ^6^Department of Imaging Physics, Delft University of Technology, Delft, Netherlands; ^7^Department of Urology, Erasmus University Medical Center, Rotterdam, Netherlands; ^8^Optical Sciences Group, Department of Science and Technology, University of Twente, Enschede, Netherlands; ^9^Biomedical Engineering and Physics, Amsterdam UMC, University of Amsterdam, Amsterdam, Netherlands; ^10^Department of Pathology, Amsterdam UMC, VU University Amsterdam, Amsterdam, Netherlands; ^11^Molecular Biophysics, Zernike Institute, University of Groningen, Groningen, Netherlands; ^12^Department of Biochemistry and Cell Biology, Faculty of Veterinary Medicine, Utrecht University, Utrecht, Netherlands; ^13^School of Pharmaceutical Sciences and Technology, Tianjin University, Tianjin, China

**Keywords:** atomic force microscopy, electrochemistry, electron microscopy, extracellular vesicles, flow cytometry, fluorescence microscopy, Raman spectrum analysis, surface plasmon resonance imaging

## Abstract

Extracellular vesicles (EVs) have great potential as biomarkers since their composition and concentration in biofluids are disease state dependent and their cargo can contain disease-related information. Large tumor-derived EVs (tdEVs, >1 μm) in blood from cancer patients are associated with poor outcome, and changes in their number can be used to monitor therapy effectiveness. Whereas, small tumor-derived EVs (<1 μm) are likely to outnumber their larger counterparts, thereby offering better statistical significance, identification and quantification of small tdEVs are more challenging. In the blood of cancer patients, a subpopulation of EVs originate from tumor cells, but these EVs are outnumbered by non-EV particles and EVs from other origin. In the Dutch NWO Perspectief Cancer-ID program, we developed and evaluated detection and characterization techniques to distinguish EVs from non-EV particles and other EVs. Despite low signal amplitudes, we identified characteristics of these small tdEVs that may enable the enumeration of small tdEVs and extract relevant information. The insights obtained from Cancer-ID can help to explore the full potential of tdEVs in the clinic.

## Introduction

Extracellular vesicles (EVs) are cell-derived particles with a phospholipid membrane. Because the membrane composition and content of EVs reflect the origin and state of the parental cells, EVs have become promising disease biomarkers (1–4). Participants from eight universities and 21 companies, who collaborate in the Dutch NWO Perspectief program Cancer-ID, aim to develop and evaluate technology to detect tumor-derived EVs (tdEVs) in blood as biomarker for cancer.

**Figure 1:**
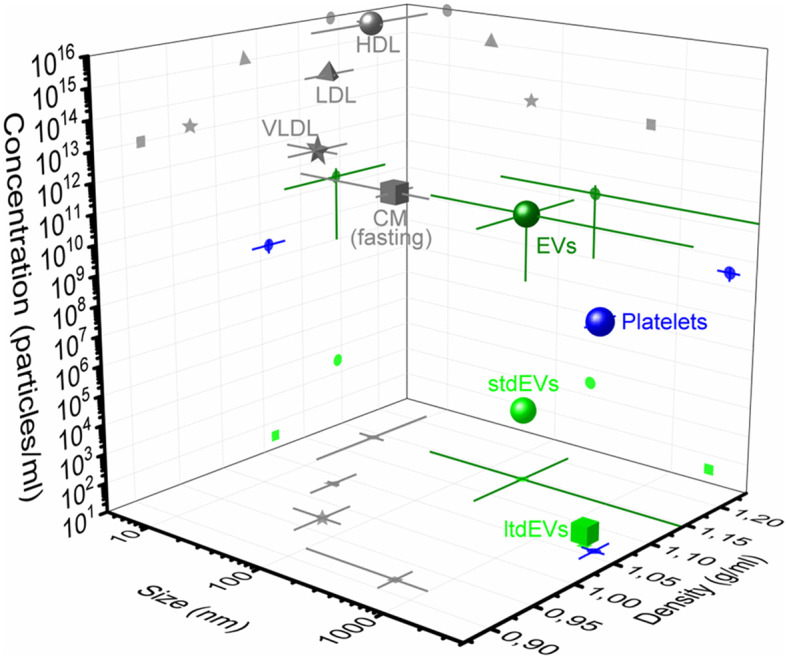
Concentration, size, and density of plasma particles. 3D representation of concentration, size, and density of extracellular vesicles (dark green circle), platelets (blue), and the high-density lipoproteins (HDLs, gray circle), low-density lipoproteins (LDL, gray triangle), very low-density lipoproteins (VLDLs, gray star), and chylomicrons (CM, gray square) during fasting in blood. The average and standard deviation (lines) of the three parameters are indicated in the figure. Values are derived from the literature (5, 7, 11). The frequency of the large tumor-derived extracellular vesicles (ltdEVs, light green circle) determined in the Cancer-ID program and the small tdEVs (stdEVs, light green square) estimated using the frequency of ltdEVs (8–10).

Throughout the project, two main challenges involved in the detection of EVs in blood became apparent. First, EV detection is hampered because EVs are outnumbered by the presence of non-EV particles in blood, like soluble proteins and lipoprotein particles at the low end of the EV size and density range, and platelets at the high end of the EV size and density range (5–7). Moreover, the concentration of larger lipoproteins, such as chylomicrons, depends on food intake, thereby emphasizing the need to discriminate EVs from other such particles. To illustrate this challenge, we determined that 1 ml of human blood of metastatic castration-resistant prostate cancer patients contains about 10 large (>1 μm) tdEVs (8, 9), and we extrapolated this to encompass the small tdEVs to arrive at an estimated 10^4^ tdEVs per 1 ml. Furthermore, the blood contains up to 10^16^ lipoproteins, up to 10^9^ platelets, and up to 10^11^ other EVs (5, 7, 10–12), see Figure 1. The second challenge is the heterogeneity of EVs in many aspects, including morphology (13), size (13, 14), membrane composition (8, 15–19), and refractive index (20, 21), which complicates EV isolation, detection, and enumeration.

In sum, utilization of tdEVs as cancer biomarker requires (i) the discrimination of EVs from non-EV particles, (ii) identification of their cellular origin, and/or (iii) analysis of the EV molecular content. The insight that an EV-based cancer biomarker requires the ability to detect, identify, and enumerate tdEVs among other particles' plasma is an essential Cancer-ID outcome because it defines the state-of-the-art. Therefore, we will use this definition to evaluate the 10 techniques that were developed or improved throughout the project. The project includes techniques that (i) detect single particles attached to a surface, such as atomic force microscopy (AFM), electrochemical (EC) detection, scanning electron microscopy (SEM), and transmission electron microscopy (TEM); (ii) detect an ensemble of EVs attached to a surface, such as surface plasmon resonance imaging (SPRi); (iii) detect single EVs in suspension, such as flow cytometry (FCM); or (iv) can measure either single or multiple EVs attached to a substrate or in a suspension, such as Raman microspectroscopy. The other evaluated technologies are integrated photonics lab-on-chip devices for Raman spectroscopy, hybrid AFM–SEM–Raman, and immunomagnetic epithelial cell adhesion molecule (EpCAM) enrichment followed by fluorescence microscopic (FM) detection. The evaluated techniques including key characteristics are listed in Table 1. This table also gives an estimate of tdEV throughput from each technique applied in a clinical setting, taking into consideration the vast majority of non-tdEV particles in plasma that may or may not contribute to the signal.

**Table 1:** Overview of the techniques used in Cancer-ID.

	**Method**	**References**	**§**	**Information obtained**	DL^*^ (nm)	Thr^*^ (prt/h)	T^*^ (h)^**a**^
**SURFACE**							
**Single**	TEM	(13)	Transmission Electron Microscopy	Morphology, size	30	9 × 10^3^	2 × 10^7^
	SEM	(15)	Scanning Electron Microscopy	Topography, size	50	50	4 × 10^2^
	AFM	(15)	Atomic Force Microscopy	Morphology, bending modulus	30	3 × 10^3^	50
	Raman	(15)	Integrated Photonics Lab-on-Chip Devices for Raman Spectroscopy	Chemical composition	80	100	2 × 10^3^
	Electrochemistry	(19)	AFM-SEM-Raman	Concentration, antigen expression	–	2 × 10^7^	0.5
**Bulk**	SPRi	(22)	Electrochemistry	Antigen expression	–	30 × 10^7^	3 × 10^−2, *b*^
**SUSPENSION**							
**Single**	NTA	(23)	Preparation of EV Samples	Particle size distribution	30	400	9 × 10^8^
	Raman	(18)	Raman Microspectroscopy in Suspension	Chemical composition	80	9 × 10^4^	6 × 10^7^
	FCM	(21)	Surface Plasmon Resonance Imaging	Antigen expression, refractive index	200	6 × 10^6^	4 × 10^6^
	FM	(8)	Flow Cytometry	Antigen expression	1,000	4 × 10^6^	2 × 10^11^

* DL, detection limit; Thr, throughput in total number of (generic) particles per hour; T, expected time needed to find 1 tdEV (specifically) in a typical plasma sample.

a This column clarifies the need for in situ enrichment and sensitive detection for diagnostic applications. Nonenriched techniques are expected to process a third of all particles in 1 μl individually or spread out over a flat surface before encountering 1 tdEV. Considering that the total area of all particles (lipoproteins and EVs, see Figure 1) distributed over a densely packed monolayer is ~36 cm^2^, the following assumptions were made:.

TEM: 2.2 × 2.2 μm^2^ imaging area, imaged in 1 min, with a capturing efficiency of 21%. SEM: 25 μl sample, 50 μm × 7 mm capturing area, 10% capturing efficiency, 10% detected fraction (due to sensitivity limitations), 5 min per 10 × 10 μm^2^ image. AFM: 25 μl sample, 50 μm × 7 mm capturing area, 10% capturing efficiency, 45 min per 25 × 25 μm^2^ image. Raman on surface: 25 μl sample, 50 μm × 7 mm capturing area, 10% capturing efficiency, 1% detected fraction (due to sensitivity limitations), 17 min per 30 × 30 μm^2^ image. Electrochemistry: Processing any sample takes ~30 min regardless of the number of tdEVs. ^b^SPRi: Processing time is 2 min. NTA: This technique can process 100 particles in 15 min, i.e., 10^10^ measurements have to be performed to find all tdEVs in 1 μl of plasma. Raman in suspension: ∽10^12^ measurements of 38 ms; 50% of particles fall below the detection limit. Flow cytometry: The sample must be diluted 10^9^ times to ensure that one detection event corresponds to one particle; 3 μl can be processed in 1 min; 50% of particles fall below the detection limit. Fluorescence microscopy: An area of 100 × 100 μm^2^ can be imaged in 0.2 s with an automated stage; 99% of particles fall below the detection limit.

*Please note that 200 μl of sample is needed before the 10^4^ EVs/μl limit of detection is reached*.

To compare all techniques, EVs derived from prostate cancer cell lines and EVs derived from platelet and red blood cell concentrates were distributed among the participants and measured. Based on the aforementioned requirements, we aimed to qualify the ability of a technique to (i) detect or image EVs, (ii) identify tdEVs, which involves differentiation of tdEVs from EVs and non-EV particles, and (iii) relate the measured signal or count to the concentration of tdEVs in plasma.

## Preparation of EV Samples

Two prostate cancer cell lines (PC3 and LNCaP) purchased from the American Type Culture Collection (ATCC, Manassas, VA) were used to obtain prostate-cancer-derived EVs. The cell lines were cultured at 37°C and 5% CO_2_ in Roswell Park Memorial Institute (RPMI)-1640 with l-glutamine (Lonza, Basel, Switzerland) supplemented with 10% *v*/*v* fetal bovine serum (FBS) and 1% *v*/*v* penicillin and streptomycin (Lonza). Medium was refreshed every second day. The initial cell density was 10,000 cells/cm^2^ as recommended by the ATCC. The cells were washed three times with phosphate-buffered saline (PBS; Sigma, Saint Louis, MO) when they reached 80–90% confluence. Next, FBS-free RPMI medium supplemented with 0.1% *v*/*v* penicillin and streptomycin was added to the cells. After 48 h of cell culture, the cell supernatant was collected and centrifuged for 30 min at 1,000 *g*. The supernatant was collected, and aliquots were snap frozen in liquid nitrogen and stored at −80°C.

Red blood cell concentrate (150 ml) obtained from Sanquin (Amsterdam, Netherlands) was diluted in a 1:1 ratio with filtered PBS [154 mM NaCl, 1.24 mM Na_2_HPO_4_·2H_2_O, 0.2 mM NaH_2_PO_4_·2H_2_O, pH 7.4; 0.22 μm filter (Merck Chemicals BV, Darmstadt, Germany)] and centrifuged three times for 20 min at 1,560 *g*. Platelet concentrate (100 ml) obtained from Sanquin was diluted in a 1:1 ratio with filtered PBS. Next, 40 ml acid of citrate dextrose (ACD; 0.85 M trisodiumcitrate, 0.11 M d-glucose, and 0.071 M citric acid) was added, and the suspension was centrifuged for 20 min at 800 *g*. Thereafter, the supernatant was centrifuged three times (20 min at 1,560 *g*) to ensure removal of platelets. The supernatant was collected, and aliquots of 50 μl were snap frozen in liquid nitrogen and stored at −80°C.

**Figure 2:**
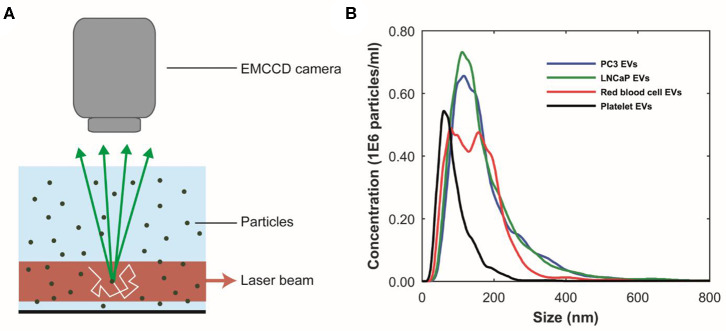
Particle size distributions of extracellular vesicle (EV) samples measured using nanoparticle tracking analysis (NTA). **(A)** Schematic representation of the NTA setup. A laser beam illuminates the particles in suspension. The light scattered by particles undergoing random motion (white arrow) is collected by a microscope objective and detected by an multiplying charge-coupled device (EMCCD) camera. The random motion of the particles under Brownian motion can be related to their size. **(B)** NTA analysis results of the PC3 EV (blue), LNCaP EV (green), red blood cell EV (red), and the platelet (black) EV samples, respectively. The bin width is 10 nm. The mean particle size and concentration in the PC3 EV sample are 172 ± 4 nm and 1E8 particles/ml, respectively. The mean particle size and concentration in the LnCaP EV sample are 167 ± 4 nm and 1E8 particles/ml, respectively. The mean particle size and concentration in the red blood cell EV sample are 148 ± 4 nm and 1E8 particles/ml, respectively. The mean particle size and concentration in the platelet EV sample are 89 ± 5 nm and 4E7 particles/ml, respectively. Because the uncertainty in the determined concentration with NTA is unknown, the determined concentration should be interpreted as an order of magnitude estimate (24). Images adapted from Lee et al. (25) and van der Pol et al. (26).

The particle size distributions of the EV samples were obtained using nanoparticle tracking analysis (NTA NS500; Nanosight, Amesbury, UK), equipped with an electron multiplying charge-coupled device (EMCCD) camera and a 405-nm diode laser (Figure 2A). Silica beads (105 nm; Microspheres–Nanospheres, Cold Spring, NY) were used to focus the microscope objective. Samples were diluted 10–2,000 times in filtered PBS to ensure that the number of particles in the field of view was below 200 per image. Of each sample, 10 videos of 30 s were captured with the camera shutter set at 33.31 ms and the camera gain set at 400. All samples were analyzed with the instrument software (NTA 2.3.0.15) using a threshold of 10, which was based on the exponential decay constant of the summed intensity histogram of all frames in each movie (MATLAB, v.7.9.0.529; Mathworks, Natick, MA).

Figure 2B shows the measured particle size distributions of the EV samples. We estimate the smallest detectable EV for NTA to be 70–90 nm (24).

## Transmission Electron Microscopy

### Cancer-ID Specific Method and Operating Principle

TEM has become the standard technique to confirm the presence of EVs in samples (27). TEM transmits electrons through sufficiently thin (<100–200 nm for biological materials) samples to make images with possibly subnanometer resolution (28). Particles from the sample are adhered to a carbon-coated formvar grid. Because EVs compete with other negatively charged particles for space on the grid, removal of soluble proteins and/or salts, for example by size exclusion chromatography (SEC) (29) and/or concentration, is required prior to incubation with EV samples. In addition, because TEM is performed in vacuum, EV samples are fixed with paraformaldehyde. After fixation and adhesion, the grid is placed on a droplet of contrast agent (uranyl acetate). A filter paper is used to remove the excess of contrast agent, and the grid is dried at room temperature (30).

**Figure 3:**
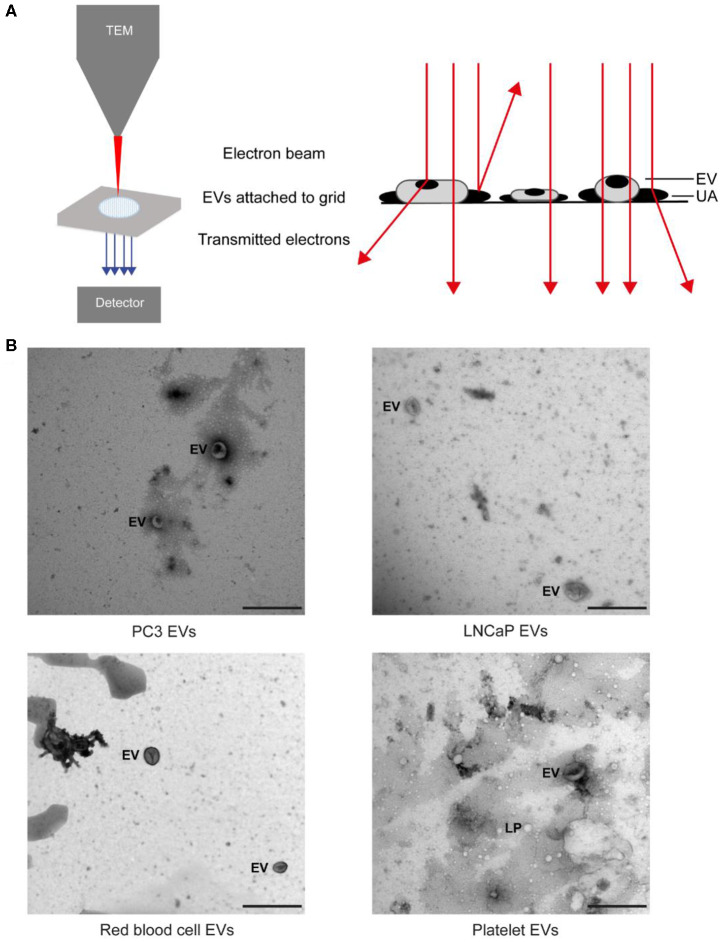
Transmission electron microscopy (TEM) of extracellular vesicle (EV) samples. **(A)** Schematic representation of TEM imaging for EV samples. The sample on a grid is exposed to an electron beam, and images are constructed based on the detected transmitted electrons. The uranyl acetate (UA) scatters electrons efficiently, which results in negative contrast. EVs and lipoproteins (LPs) have a low electron density and are seen as bright particles in a dark background (13). **(B)** TEM images of the EV samples from PC3 and LNCaP and of red blood cells and platelets after size exclusion chromatography. The scale bar corresponds to 500 nm (25).

Next, the grid is placed in the vacuum chamber of a FEI Tecnai 12 transmission electron microscope (FEI, Eindhoven, Netherlands). The sample is exposed to an electron beam, and images are constructed based on the detected transmitted electrons (Figure 3A). The contrast agent scatters electrons efficiently and stains the background more efficiently than the EVs. Consequently, EVs appear as bright particles on top of a dark background.

### EV Definition

Water, the main cargo of an EV, is evaporated upon TEM resulting in a deformation of EVs, which often appear as “cup-shaped” (31–34) or “saucer/doughnut-shaped” particles (35–37) (Figures 3C–**F**). Particles, not having water as their major component, maintain their original structure during TEM. For example, lipoproteins appear spherical, and protein aggregates have an irregular shape. Therefore, we define EVs as cup-shaped particles larger than 30 nm (13).

### Value Added by Cancer-ID

We show that TEM images taken by operator selection, the current standard within the EV field, can be used to demonstrate the presence of EVs in a sample. However, the examination of the morphology of EVs by TEM shows an operator bias in their identification (13), which may lead to “cherry picking” and emphasizes the importance of an automated and objective assessment of EV identification. Two important steps to improve the comparability and reproducibility of TEM for monitoring the quality of EV samples are (1) to take images at predefined locations and (2) provision of both close-up and wide-field images, as adopted by MISEV2018 (38).

### Relevance for Cancer Diagnostics

Although with appropriate sample preparation, TEM can image EVs down to 30 nm, the contrast of TEM images is often insufficient to distinguish EVs from similar sized non-EV particles (Figure 3B). Moreover, to identify detection markers on tdEVs, immuno-gold labeling (34) is necessary. However, the main limitation of TEM for tdEV detection is the low throughput because of the scarcity of tdEVs among other abundant particles in plasma (see Table 1). Therefore, TEM can be used to evaluate the quality and presence of EVs but is not a relevant technique for identification of tdEVs in plasma samples.

## Scanning Electron Microscopy

### Cancer-ID Specific Method and Operating Principle

EV samples are fixed in paraformaldehyde, followed by gradual dehydration from 70 to 100% ethanol in water with a 10% concentration increment step every 5–10 min. Subsequently, chemical drying of the sample can be achieved using 1:1 hexamethyldisilazane (HMDS) in ethanol for 3–5 min, followed by 100% HMDS for 3–5 min more. EVs are dehydrated and dried to maintain their morphological and surface features with minimal deformation in the vacuum chamber of the SEM (39, 40). EV samples are coated with gold to increase the image contrast and avoid surface charging. Furthermore, the sample must be placed on a conductive substrate during imaging. The entire procedure is conducted at room temperature.

**Figure 4:**
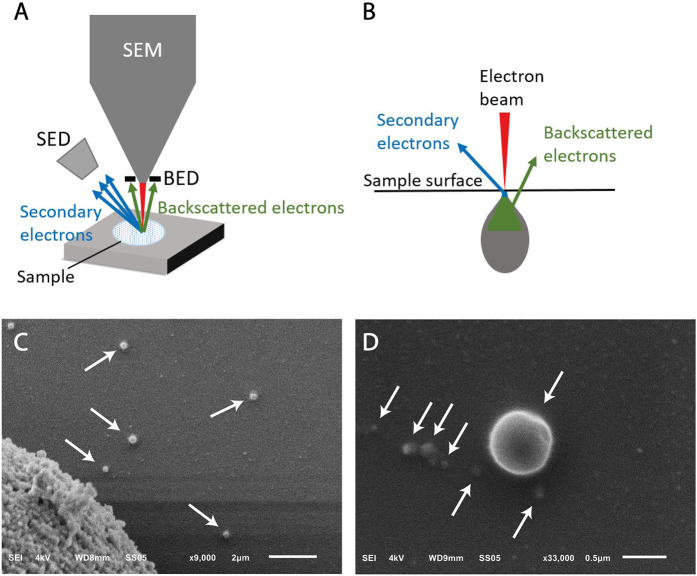
Scanning electron microscopy (SEM) of extracellular vesicle (EV) samples. **(A)** Schematic representation of a SEM setup. SED, secondary electron detector; BED, backscatter electron detector. **(B)** The sample is illuminated by the electron beam. Electrons interact with the sample at different depths, resulting in emitted electrons from the surface (secondary electrons) and from deeper layers (backscattered electrons). **(C)** SEM image of LNCaP EVs indicated by arrows. The large object in the left lower corner is part of a LNCaP cell floating in the cell supernatant and was imaged to show that the contrast of EVs is similar to cells. The scale bar represents 2 μm. **(D)** Higher magnification allows imaging of smaller particles, possibly EVs, with lower contrast. The scale bar represents 500 nm.

In SEM imaging, a focused beam of electrons scans the surface of a sample interacting with all atoms in the sample (Figures 4A, B). Detection of the secondary electrons, originating from the outer layers of the sample, enables to visualize the topography of a sample. The amount of backscattered electrons, originating from the deeper layers of the sample, is associated with the atomic number of the atoms in the sample.

### EV Definition

Since the LNCaP EV sample is derived from cell culture, we do not expect particles like lipoproteins to be present in this sample. Figure 4B shows round particles (white arrows) in lower and higher magnification, which we define as EVs.

### Value Added by Cancer-ID

We show that cells and EVs captured on functionalized substrates and in solution can be imaged by SEM.

### Relevance for Cancer Diagnostics

SEM can be used to visualize the topography of tdEVs, as small as 50 nm, but is unable to discriminate EVs from non-EV particles with a similar morphology. In order to confirm the nature of the particles, immunogold labeling or correlative techniques are required such as AFM, Raman, or fluorescence imaging. Cryo-TEM and cryo-SEM have been suggested superior techniques in retaining the EV morphology when compared to TEM and SEM because of the effect of fixation and air dehydration in the vacuum chamber (34); however, our results (Figure 4) support that gradual dehydration of EV samples in ethanol series prior to SEM imaging allows the maintenance of EV morphology. The main limitation of SEM similarly to TEM is the low throughput, as a large area needs to be processed before tdEVs are encountered (see Table 1). Therefore, SEM is not a relevant technique for the detection of tdEVs in plasma samples.

## Atomic Force Microscopy

### Cancer-ID Specific Method and Operating Principle

**Figure 5:**
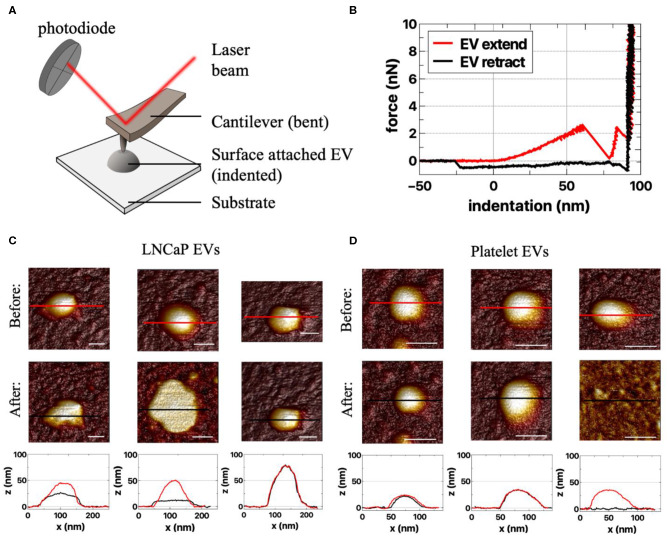
Atomic force microscopy (AFM) of extracellular vesicle (EV) samples. **(A)** Schematic representation of the AFM setup. In AFM, a cantilever interacts with the sample, and the reflected laser beam is detected by a photodiode (45). The experiments are performed in liquid (not depicted). **(B)** Example of force–indentation curves (distance *z*) of the extend and retract response on an EV (45). AFM images of responses of **(C)** LNCaP EVs and **(D)** platelet EVs to an applied force before (first row) and after indentation (second row). Both the LNCaP and the platelet EVs can change shape upon indentation. The different responses are illustrated by the cross-sections (bottom row), taken at the indicated spots in the corresponding AFM images above (red, before indentation; black, after indentation). Scale bars represent 50 nm.

EVs are added onto a poly-l-lysine-coated coverslip (41–44). Next, the sample chamber is filled with filtered PBS (0.2 μm filter; VWR International, Radnor, PA) and placed on the AFM. During AFM imaging, a cantilever with a nanometer-sized tip probes the sample surface (Figure 5A) (45). Deflection of the cantilever is measured with a laser and photodiode. AFM images are acquired in PeakForce Tapping mode on a Bruker Bioscope catalyst setup using minimal imaging force providing information about the topography of the samples surface. Mechanical properties can be obtained by applying a defined force perpendicular to the surface (indentation), providing force–indentation curves, as presented in Figure 5B.

### EV Definition

With AFM, we characterize an EV as a particle of at least 25 nm in height with a spherical shape. Aggregates typically have a nonspherical shape and therefore can be excluded. The nanoindentation response is used to identify single EVs (42, 44). A typical indentation curve is characterized by a (close-to) linear initial increase in force followed by a softening and finally bilayer pinching close to the substrate (Figure 5B, red curve).

### Value Added by Cancer-ID

Unique characteristics, like deformability, of tdEVs compared to EVs of other origin still need to be explored. Examples of AFM measurements of LNCaP EVs and platelet EVs are shown in Figures 5C,**D**. Importantly, it should be noted that AFM imaging *per se* is not distinguishing between EVs and lipoproteins. Therefore, a good purification protocol is necessary (combining gradient- and size-based isolation methods) in order to assure only EVs are present.

### Relevance for Cancer Diagnostics

Because of the nanometer position sensitivity and subpiconewton force sensitivity, AFM can be used to determine the topography, morphology, and mechanical characteristics of single EVs, and differences between EVs of different origins can be investigated (42, 44). AFM is a low-throughput technique since only several particles can be observed at a time. For the moment AFM has not been shown to be a suitable technique for tdEV identification and enumeration in plasma samples (see Table 1).

## Raman Microspectroscopy in Suspension

### Cancer-ID Specific Method and Operating Principle

**Figure 6:**
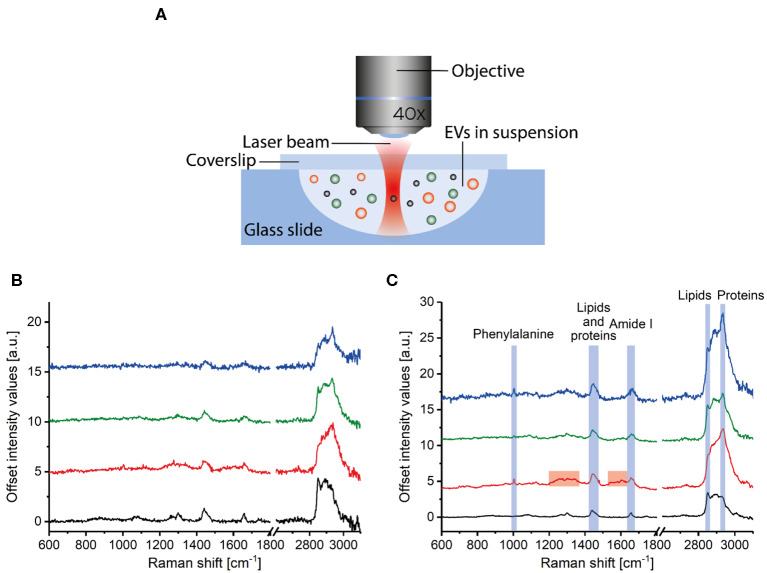
Raman spectroscopy of extracellular vesicle (EV) samples. **(A)** Particles in suspension are loaded in a well glass slide that is mounted under a microscope objective. Incident light illuminates the sample, and both Raman and Rayleigh light are backscattered, collected by the lens, and detected by a spectrograph. Raman spectra corresponding to **(B)** single and **(C)** multiple PC3 EVs (blue), LNCaP EVs (green), red blood cell EVs (red), and lipoproteins in plasma (black). **(A)** is adapted from Enciso-Martinez et al. (18).

EV samples are diluted in PBS to a concentration of ~10^9^ particles/ml (as measured by NTA) and placed on a well glass slide, covered with a glass cover slip, and sealed with glue. Next, the glass slide is placed under the microscope objective (Figure 6A). A Raman optical tweezers [home-built system as described in Enciso-Martinez et al. (18)] is used to (i) trap single particles diffusing near the high intensity part of the focus (Figure 6A) and (ii) detect both Rayleigh and Raman scattered photons synchronously. The trapping of a single particle is detected by Rayleigh scattering, and the corresponding Raman spectrum discloses the chemical composition (18, 46).

### EV Definition

The Raman spectra of submicrometer particles in biofluids have distinct spectral features depending on the nature of the particle or the source of EVs.

### Value Added by Cancer-ID

The procedure to trap, release, and acquire sequentially the spectrum of single EVs in the focal volume is automated (18). Furthermore, EVs can be distinguished from lipoproteins and EVs from different sources, like PC3 EVs, LNCaP EVs, and red blood cell EVs. EVs show distinctive peaks at 1,004 and 1,607 cm^−1^ (phenylalanine) and a larger protein contribution at 2,811–3,023 cm^−1^ than lipoproteins (Figures 6B,**C**) (25, 46). The Raman spectrum of red blood cell EVs is different from PC3 EVs and LNCaP EVs around 1,200–1,385 cm^−1^ and 1,510–1,631 cm^−1^ (46). Further classification of EVs and lipoproteins was achieved by multivariate analysis and convolutional neural networks analysis (25, 47).

### Relevance for Cancer Diagnostics

Differences in chemical composition are shown between EVs and lipoproteins, and tdEVs compared to red blood cell EVs. However, a limitation of Raman is the throughput. As an example, a typical acquisition time per EV is 1 s (18). Hence, it has become clear that enrichment of tdEVs is needed prior to Raman analysis. Nevertheless, spontaneous Raman spectroscopy provides information on the chemical composition of single or multiple EVs in solution or on a surface in a noninvasive and label-free manner (18, 25, 46, 48–52).

## Integrated Photonics Lab-On-Chip Devices for Raman Spectroscopy

### Cancer-ID Specific Method and Operating Principle

**Figure 7:**
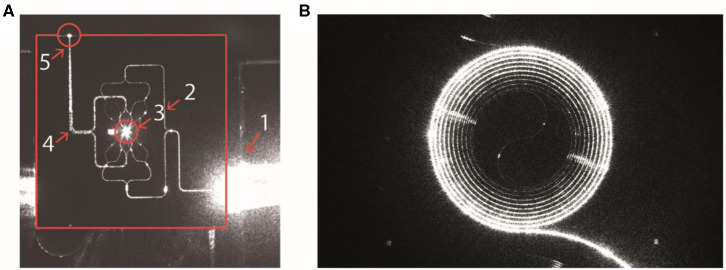
Integrated photonics based lab-on-a-chip Raman spectroscopy. **(A)** Device type 1: Camera image of a device with 16 waveguides for trapping and four waveguides for detection. The device is actuated with light from an input fiber that is embedded in a fiber array unit (FAU) at the lower right-hand side. The various structures light up as a result of light scattering, causing some saturation of the camera. The solid red lines indicate the chip edges. 1, FAU; 2, excitation-waveguide circuitry; 3, microfluidic bath with the central trapping region; 4, detection-waveguide circuitry; 5, light from the trap that is coupled out by the detection waveguides. Here, the detection waveguides collect light as a result of direct illumination and scattering (from Loozen et al. (53)). **(B)** Device type 2: Spiral waveguide with the Raman pump light traveling inside the waveguide. The Raman signal is (partially) scattered back into the waveguide and collected at the front entrance. Reproduced with permission from Lee (54).

Two types of lab-on-chip devices were developed by Cancer-ID and fabricated in the cleanroom of the MESA+ Institute of Technology. From a technological perspective, Cancer-ID exploits the possibility of lab-on-a-chip devices to localize light in ways that are impossible with traditional optics. For example, compared to optical trapping using a microscope objective (*Raman Microspectroscopy in Suspension*), we expect that combining multiple beams will result in higher field gradients and therefore trapping of smaller single EVs. To proof the principle, device type 1 contains multiple waveguides, each of which emits a narrow beam of light towards the center of a fluidic microbath, as shown in Figure 7A. The resulting multiple beams combine coherently to form an interference pattern with multiple spots of high light intensity, each serving as an optical trap sufficiently strong to trap single submicrometer particles near the microbath center. The same concentrated light induces a Raman spectrum from the trapped particle for label-free identification. To increase the throughput, the well may be replaced by a flow cell in future versions.

To increase throughput compared to optical trapping using a microscope objective (section *Raman Microspectroscopy in Suspension*), device type 2 combines an enrichment step with the simultaneous detection of Rayleigh and Raman scattered light from multiple EVs. EVs in suspension bind to antibodies at the surface of a spiral waveguide, which is placed at the bottom of a microfluidic channel as shown in Figure 7B (54). A laser field propagates inside the waveguide and produces an evanescent field that probes the attached EVs simultaneously. The EVs will scatter some of this light with characteristic Raman shifts. A significant portion of this light re-enters the waveguide and can be collected from the entrance through the same objective that launched the excitation light.

### EV Definition

An EV is identified based on the acquired Raman spectrum of the trapped particle. The obtained spectra may be cross-referenced with EV spectra already acquired with standard spontaneous Raman tweezers (*Raman Microspectroscopy in Suspension*). Furthermore, using device type 2, EVs are bound to the surface of a spiral waveguide by a specific antibody.

### Value Added by Cancer-ID

Both device types are still under development. So the throughput and detection limit remain to be determined. In device type 1, integration of the multiple beam trap with a microfluidic channel opens new possibilities of controlled particle delivery to the trap and particle sorting with pressure-driven flow, which may allow the detection of smaller EVs. In device type 2, specific capture of tdEVs from the plasma is possible by the use of antibodies coated on the surface of a spiral waveguide using the chemistry used in *AFM-SEM-Raman* and *Electrochemistry*.

### Relevance for Cancer Diagnostics

Based on the differences in chemical composition, tdEVs can be distinguished from non-EV particles like lipoproteins and EVs from other origin. Furthermore, enrichment can be achieved by the use of antibodies bound to the surface of a waveguide. Raman spectroscopy of EVs provides information on the chemical composition of single or multiple EVs in a noninvasive and label-free manner and may be simplified using integrated photonics lab-on-a-chip devices. The analysis time per particle remains to be measured before estimating the tdEV throughput and potential of the specific technique.

## AFM–SEM–RAMAN

### Cancer-ID Specific Method and Operating Principle

**Figure 8:**
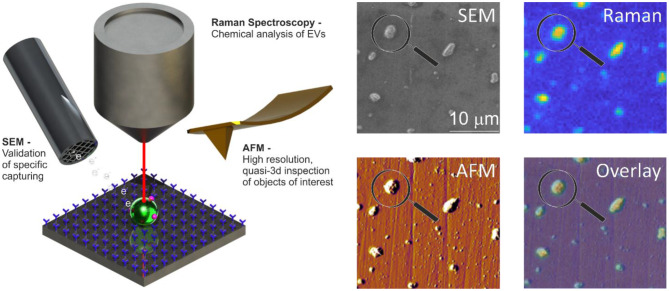
Atomic force microscopy (AFM), scanning electron microscopy (SEM), and Raman spectroscopy of extracellular vesicle (EV) sample. Schematic representation of the system: antibody-functionalized stainless-steel substrate examined with SEM, AFM, and Raman for correlated multimodal analysis of individual EVs. Image adapted from Beekman et al. (15).

The surface of stainless-steel substrates is modified with a carboxydecyl phosphonic acid monolayer to covalently link anti-EpCAM antibodies to the substrate (Figure 8) (55). EVs are incubated in poly(dimethylsiloxane) (PDMS) microchannels. The microchannels are washed to remove nonspecifically bound material. Next, EVs are incubated with paraformaldehyde in PBS for 15 min. The PDMS is removed by immersion in deionized water, 70% ethanol in water, and finally 100% ethanol for 5 min each step. Dehydration of tdEVs was followed by overnight drying. Alignment markers are embedded on the stainless-steel substrate by injecting patterned microfluidic channels with cyanoacrylate glue. The microscale alignment markers facilitate retracing individual EVs in the sample stages of the AFM (MFP-3D, Asylum Research, Wiesbaden, Germany), SEM (JEOL JSM-6610LA Analytical SEM, JEOL, Nieuw-Vennep, Netherlands), and Raman microspectroscopy ]home-built system as described in Beekman et al. (15)]. SEM is used here to select regions of interest and confirm that the surface is successfully functionalized based on the attachment of EVs (15).

### EV Definition

EVs are identified by SEM and a Raman spectrum with lipid-protein peaks (2,811–3,023 cm^−1^) characteristic for EVs. The functionalization of the substrate ensures that the EVs are of epithelial cell origin permitting the determination of the mechanical characteristics, like deformability, of the tdEVs by AFM.

### Value Added by Cancer-ID

The use of only one technique is often insufficient to identify and characterize EVs, as discussed in the previous sections (38). For example, both EVs and lipoproteins appear to be spherical by SEM. By combining SEM with AFM and Raman, we measure characteristics like size, chemical composition, and deformability to add certainty to the identification of tdEVs (15).

### Relevance for Cancer Diagnostics

Using a combination of AFM, SEM, and Raman and the capture of tdEVs to a functionalized surface helps to distinguish EVs from non-EV particles and adds certainty to the origin of the EV.

In principle, this platform does not require distinguishing tdEVs from other species since enrichment is done by the functionalized surface (as assumed in Table 1). Since SEM measurements are faster than AFM or Raman, SEM was used for initial confirmation of tdEV presence on a chip; after enrichment, 1,000 tdEVs (of >100 nm) can be imaged in 1 h. Since AFM detects the more abundant much smaller particles (>30 nm) as compared to SEM (>100 nm), the fact that AFM is slower in terms of imaged square micrometer per unit time, is offset by a greater number of observed tdEV per imaged square micrometer, such that 1,000 tdEVs can be imaged in 2 h. For Raman, detection of 1,000 tdEVs would require about 100 measurements of 17 min each followed by several days of data processing.

## Electrochemistry

### Cancer-ID Specific Method and Operating Principle

**Figure 9:**
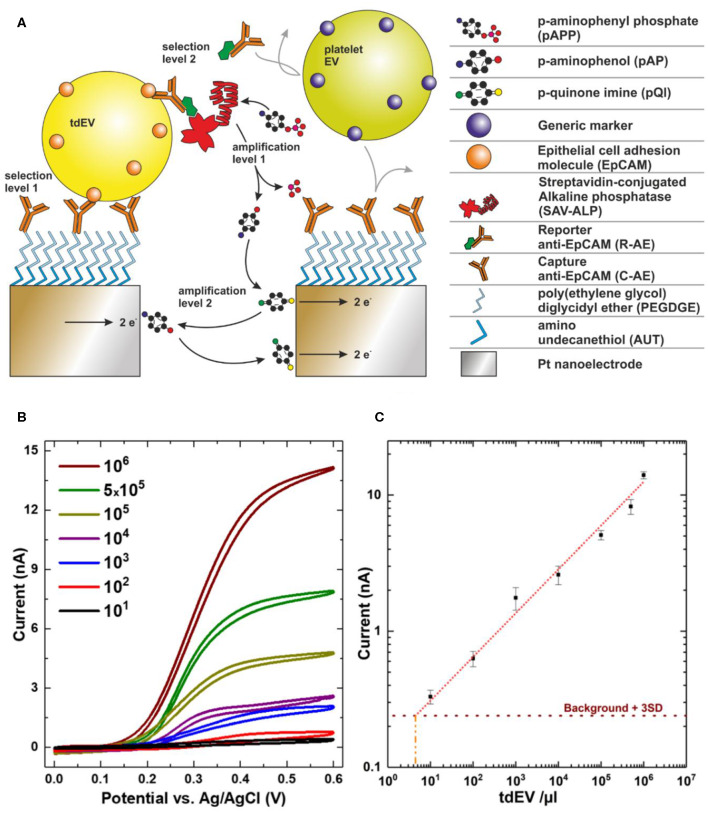
Electrochemical detection of extracellular vesicle (EV) samples. **(A)** Scheme showing selective capture and *in-situ* labeling of EVs followed by enzymatic amplification of redox species and redox cycling. **(B)** Cyclic voltammograms recorded for a very wide range of EV concentrations. **(C)** Recorded current at 0.4 V for varying concentrations showing linear response over six orders of magnitude. Image adapted from Mathew et al. (19).

Interdigitated nanoelectrodes (nIDEs), fabricated in the cleanroom of MESA+ Institute for Nanotechnology, are surface-modified with poly(ethylene glycol) diglycidyl ether to form an amine-reactive antifouling layer (Figure 9A) (56). Antibodies against EpCAM (VU1D9 clone) are covalently linked to this layer and the remainder of the surface blocked with bovine serum albumin (BSA). EV samples are introduced onto the device to allow binding to the electrodes. After incubation, a biotinylated reporter anti-EpCAM is introduced. The biotin moiety conjugates to streptavidin coupled to alkaline phosphatase (ALP). ALP, only present on EpCAM-positive particles, converts an electrochemically inert molecule (para-aminophenyl phosphate) into a redox-active species (para-aminophenol), to yield a first amplification phase. Next, the para-aminophenol undergoes redox cycling, providing a second amplification phase.

### EV Definition

An increase in the redox current upon binding of particles to the nIDEs defines the presence of EVs. EVs from different species can be distinguished from each other by employing targeted antibodies, yielding a very high selectivity. For example, the signal from platelet EVs did not vary from the background signal, whereas the introduction of LNCaP EVs markedly increased the signal (Figure 9B) (19).

### Value Added by Cancer-ID

This new and sensitive technique was developed by Cancer-ID in collaboration with researchers from the NanoElectronics group at University of Twente, Netherlands. Several examples of sensitive integrated systems for (td)EV detection exist (57–60) (Lorencova). A unique feature of the technique discussed here is the ability to detect a low concentration of EVs with a low antigen expression. The linear response covers a broad range of concentrations, which largely overlaps with concentrations of tdEVs in patient blood.

### Relevance for Cancer Diagnostics

Using electrochemistry, tdEVs can be discriminated from non-EV particles and EVs from other origin based on the expression of EpCAM. A dilution series of LNCaP EVs in PBS showed a linear response ranging from 5 × 10^3^-10^9^ tdEVs/ml (Figure 9C) (19), which overlaps with the expected tdEV concentration in plasma (10), showing that this technique is promising to identify, count, and characterize tdEVs in the range of clinical samples. Evaluation of the technique with plasma patient samples and association of the readout with clinical outcome remain to be tested.

The functionalized device is incubated with tdEV-containing sample and subsequently with reporter antibodies and redox mediator. In the experiments performed in the paper, these incubations were done over excessively long periods (2.5 h in total) to maximize the efficiency but, once optimized, can probably be performed several minutes to 1 h. The cyclic voltammetry measurements were performed in 20 min, regardless of the concentration of tdEVs (5 × 10^3^-10^9^/ml of sample). Using patient plasma samples rather than cell culture medium may increase the background signal, thereby reducing the sensitivity of the technique. Nevertheless, compared to other techniques, electrochemical methods hold great promise to be applied in a clinical setting because of the high throughput.

## Surface Plasmon Resonance Imaging

### Cancer-ID Specific Method and Operating Principle

**Figure 10:**
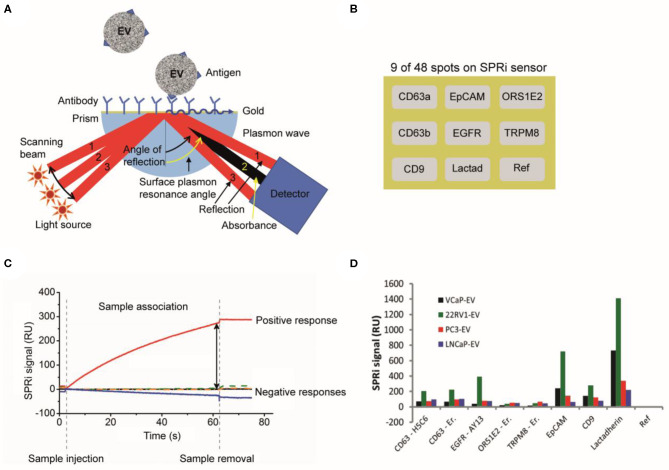
Surface plasmon resonance imaging (SPRi) analysis of extracellular vesicles (EVs). **(A)** Schematic of a SPRi setup. A SPRi signal is generated when the sensor surface is illuminated at various angles with light and surface plasmons are excited (61). The resonance angle, specific angle (beam 2) where maximum plasmon excitation and minimal internal reflection occurs (62), depends on the refractive index contrast near the interface in the evanescent field. **(B)** The 48 spots on the SPRi sensor surface can be coated with different antibodies. In this example, only nine antibody-coated spots on the SPRi sensor surface are shown. **(C)** An EV sample is exposed to the SPRi sensor and measured for 60 min. The attachment of an ensemble of EVs to a specific antibody spot causes a change in the refractive index and generates a SPRi signal over time (16). **(D)** The SPRi signals after incubation with four prostate-cancer-derived EV samples are shown. The two CD63 clones show the same results for all samples. All samples are slightly positive for CD63, EGFR, and CD9. A higher positivity is seen for EpCAM and lactadherin. The SPRi signals for the 22RV1-EV sample are higher compared to the other samples (16).

The surface of a SPRi sensor is coated with a conductive gold layer and a 3D hydrogel-like layer to reduce nonspecific binding of non-EV particles to the surface (Figure 10A). Antibodies are printed on 48 spots on the sensor (Figure 10B), including isotype controls and a control (PBS) to correct for dissociation and nonspecific binding (16). Next, the surface is washed and deactivated by incubation with 2-amino ethanol followed by BSA. After an EV sample is exposed to the sensor, EVs bind to the antibody-coated sensor spot, which increases the refractive index near the sensor surface. This increase in refractive index is measured in time using the angle scanning principle of the IBIS MX96 instrument (IBIS Technologies, Enschede, Netherlands) and corresponds to the number of particles captured on the spot (Figure 10C).

### EV Definition

With SPRi, EVs are identified based on their antigen exposure. EVs bind to antibodies printed on the sensor, e.g., anti-CD9, anti-CD63, antiepidermal growth factor receptor (anti-EGFR), anti-EpCAM, antiolfactory receptor 51E2 (anti-OR51E2), transient receptor potential cation channel subfamily M member 8 (TRPM8), and lactadherin, see Figure 10D. SPRi detects a difference in response on the antibody spots between EV samples derived from different cell lines.

### Value Added by Cancer-ID

Characterization of EVs by SPRi, using the IBIS MX96, revealed the ability to detect cell surface antigens present at relatively low antigen densities compared to cells, as their presence could not be detected by flow cytometry (16).

### Relevance for Cancer Diagnostics

SPRi can be used to distinguish tdEVs from non-EV particles and EVs derived from other cells based on the antigen expression. The IBIS MX96 is able to detect antigens present at a low density on EVs compared to cells (16). SPRi has superior sensitivity when compared to flow cytometry (16) and ELISA (63). However, the required EV concentration to perform the herein reported measurements is high (2 × 108 EVs/mL), and not within the range of the expected tdEV frequency in plasma patient samples. A different configuration (nano-plasmonic exosome (nPLEX) assay) has been suggested by Im et al. to increase the throughput and make feasible the detection of tdEVs in ascites of ovarian cancer patient samples (63).

## Flow Cytometry

### Cancer-ID Specific Method and Operating Principle

EV samples are diluted in PBS (21-031-CV; Corning, Corning, NY) to prevent swarm detection (64) and stained with fluorescently labeled antibodies. Antibody aggregates are removed by centrifugation prior to use. The “antibody supernatant” is added to the EV sample followed by a 2-h incubation step, which is stopped by diluting the incubated sample with PBS.

**Figure 11:**
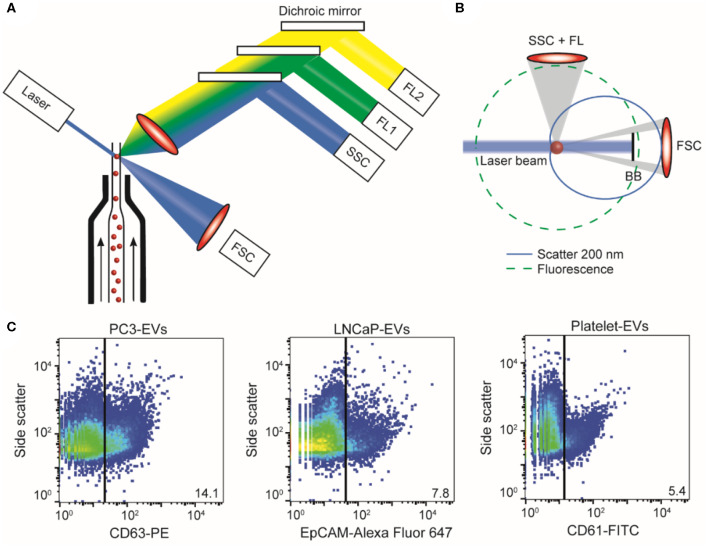
Flow cytometry of extracellular vesicle (EV) samples. **(A)** In flow cytometry, a single particle suspension is hydrodynamically focused with sheath fluid (arrows) to intersect a laser. Light coming from the particle is collected by a forward scatter detector (FSC), a side scatter detector (SSC), and multiple fluorescence detectors (FL1, FL2, etc.). **(B)** Fluorescence (green dashed line) is isotropic and can be used to determine antigen expression and cellular origin. Scatter (blue solid line) has an angular distribution that depends on the size and refractive index of the particle (here, 200 nm polystyrene). Knowledge of the flow cytometer collection angles and Mie theory allows derivation of particle size and refractive index from the measured scatter signals (14, 20). **(C)** Scatter plots of side scatter vs. fluorescence for the PC3 EV sample stained with CD63-PE (left), the LNCaP EV sample stained with EpCAM-Alexa Fluor 647 (center), and the platelet EV sample stained with CD61-FITC (right). In PC3 EV sample, 14.1% was found to be positive for the EV marker CD63; in the LNCaP EV sample, 7.8% was found to be positive for cell surface epithelial marker EpCAM; and in the platelet EV sample, 5.4% of the particles was found to be positive for CD61. BB, blocker bar; FL, fluorescence.

In a flow cytometer, the sample is hydrodynamically focused with sheath fluid to intersect a laser beam (Figure 11A). Scattered light and fluorescence from the particle are collected by a forward scatter detector, a side scatter detector, and multiple fluorescence detectors (65) (Figure 11B). The measured scatter and fluorescent signals per particle can be represented and analyzed using scatter plots as shown in Figure 11C. In the works referenced here, samples were analyzed on an A60-Micro (Apogee, Hertfordshire, UK).

### EV Definition

EV identification by FCM is commonly based on the expression of one or more antigens, which are detected using fluorescent immunostaining. Recently, we found that the refractive index of particles can be used as an additional parameter to distinguish EVs from lipoproteins (21). We therefore define an EV as a particle that expresses detectable levels of one or more antigens and has a refractive index <1.42.

### Value Added by Cancer-ID

Within Cancer-ID, a technology to determine the size and refractive index of submicrometer particles was partly developed, evaluated, and used to find new applications. Based on refractive index, for example, EVs can be differentiated from lipoproteins without antibody labeling (21). Refractive index determination was used to show that generic EV dyes, which are commonly used to label EVs in FCM measurements, do not label all EVs and do label non-EV particles (17). The combination of antibody labeling and refractive index determination could be used to increase specificity of EV detection. Furthermore, the side scatter sensitivity of a conventional flow cytometer was improved 30-fold by systematically modifying the hardware, and a method was developed to quantify the scatter sensitivity of a flow cytometer.

### Relevance for Cancer Diagnostics

FCM measures light scattering and fluorescence from thousands of individual particles per second. Although detection of the smallest single EVs is possible (66), only the most sensitive commercial flow cytometers are able to detect EVs with a diameter <200 nm (67). Based on the combination of an antibody and the refractive index, it is possible to discriminate tdEVs from lipoproteins and EVs from other origin. However, plasma samples are typically prediluted 10–100 times before measurements to prevent swarm detection (as assumed in Table 1). This dilution means that the detection of the few tdEVs that might be present in the plasma sample is impossible.

However, FCM provides information on the concentration, cellular origin and biochemical composition, size, and refractive index of single EVs (14, 20, 68).

## Immunomagnetic EpCAM Enrichment Followed by Fluorescence Microscopic Detection

### Cancer-ID Specific Method and Operating Principle

**Figure 12:**
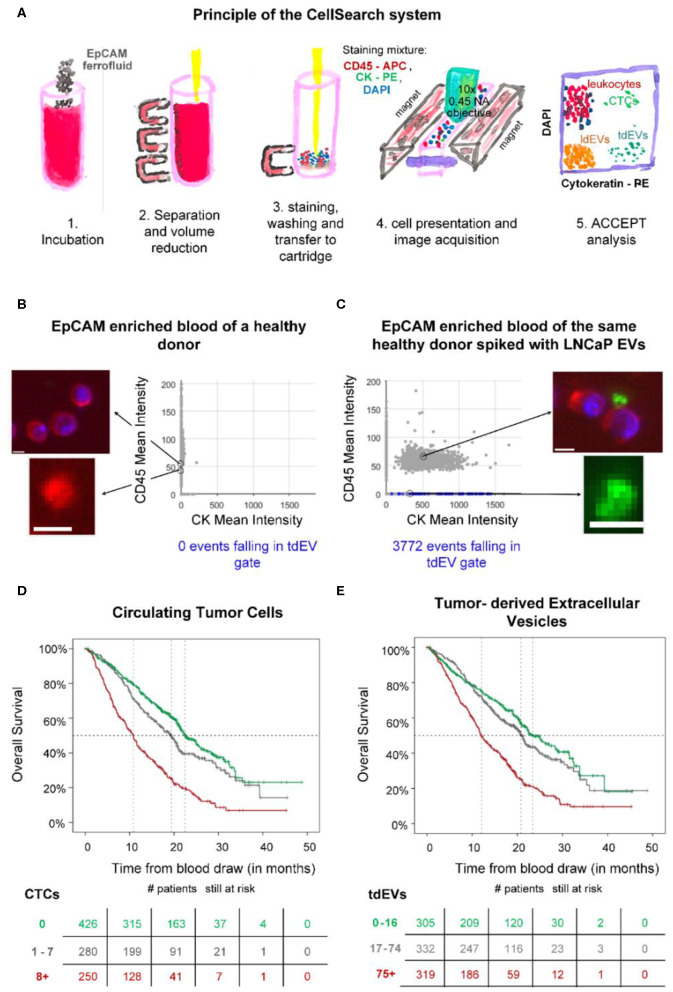
Epithelial cell adhesion molecule (EpCAM) immunomagnetic enrichment and fluorescence microscopic (FM) detection of extracellular vesicle (EV) samples. **(A)** Principle of the CellSearch system. ACCEPT analysis of two CellSearch cartridges corresponding to EpCAM-enriched blood sample of a healthy donor **(B)** without and **(C)** with LNCaP EVs spiked. CD45 is depicted in red, CK in green, and 4′,6-diamidino-2-phenylindole (DAPI) in blue. The objects falling in the applied tdEV gate are depicted as blue dots in the scatter plots of CD45 mean intensity vs. CK mean intensity. The other particles are shown as gray dots. Thumbnail examples of four objects are shown. The CD45^+^ and CK^+^ particles are attached to the leukocytes, as illustrated. Scale bars indicate 6.4 μm. **(D,E)** show Kaplan Meier plots of overall survival of 956 metastatic colorectal, prostate, breast, and nonsmall cell lung cancer patients. Patients were grouped based on their **(D)** circulating tumor cells (CTC) or **(E)** tumor-derived EV (tdEV) counts demonstrating the equivalent prognostic power of CTCs and tdEVs (9).

Blood of individuals is collected in CellSave blood collection tubes (Menarini, Huntingdon Valley, PA). After centrifugation of 7.5 mL of the blood for 10 min at 800 *g*, the sample is placed in the CellTracks Autoprep (Menarini, Huntingdon Valley, PA). The Autoprep aspirates and discards the plasma, whereas the blood cell fraction is incubated with anti-EpCAM (VU1D9 clone) ferrofluid (Figure 12A, step 1). The particles (cells and EVs) bound to the ferrofluid are separated from the rest of the blood by the application of magnetic forces (step 2). Following the immunomagnetic isolation, EpCAM-enriched particles are stained with the nuclear dye 4′,6-diamidino-2-phenylindole (DAPI) and fluorophore-conjugated antibodies recognizing the epithelial specific cytokeratins 8, 18, and 19 (CK-PE) and the leukocyte-specific marker CD45 (CD45-APC) (step 3). The stained sample is loaded in a cartridge and placed between two magnets configured in such a way that all stained EpCAM^+^-enriched particles homogeneously align on the glass slide on the surface of the cartridge (step 4). The cartridge is scanned using the CellTracks Analyzer II (Menarini, Huntingdon Valley, PA), a fluorescence microscope equipped with a 10 × 0.45 NA objective (step 4). The images are analyzed using the open-source ACCEPT software to identify circulating tumor cells (CTCs), tdEVs, leukocytes, and leukocyte derived EVs (69) (step 5).

### EV Definition

tdEVs are defined as EpCAM+, CK+, DAPI–, and CD45– particles. A gate for their automated enumeration from the CellSearch image data sets has previously been reported (8).

### Value Added by Cancer-ID

In the frames of the Cancer-ID program, we reanalyzed digitally stored FM image data sets of retrospective clinical studies acquired after EpCAM enrichment. Our results suggest that large tdEVs (>1 μm), coisolated with CTCs, are negatively associated with the overall survival of metastatic prostate, colorectal, breast, and nonsmall cell lung cancer patients in a similar way as CTCs (Figure 12) (9) and could contribute in monitoring the disease and assessing therapeutic efficacy. However, the existing technique was developed for the detection of CTCs and eliminates the detection of smaller tdEVs or tdEVs with low antigen density even if they have been isolated by the anti-EpCAM ferrofluid.

To evaluate whether tdEVs from a model cancer cell line can be isolated using the CellSearch assay, two samples were used as a positive and negative control of the technique. Two blood samples of 7.5 ml, collected in CellSave tubes and drawn from an anonymous healthy individual, were provided by the TNW-ECTM-donor services (University of Twente, Enschede, Netherlands). Both samples were processed with the CellSearch system; however, the one sample remained intact without the addition of any EVs (negative control), whereas the other one was spiked with EVs produced from the EpCAM^+^ LNCaP prostate cancer cell line (positive control). The application of a tdEV gate resulted in 0 events in the negative control and in 3,772 events in the positive control (Figures 12B,C).

This study was carried out in accordance with the recommendations of Dutch regulations. The protocol was approved by the Medical Ethical Assessment Committee Twente (METC Twente). The subject gave written informed consent in accordance with the Declaration of Helsinki.

### Relevance for Cancer Diagnostics

The CellSearch system can be used to enrich CTCs and tdEVs based on their EpCAM expression, as EpCAM is not expected to be present on cells and EVs in blood of healthy individuals (70, 71). However, tdEVs isolated by the CellSearch system are limited to the larger EVs (>1 μm), as the technique was designed to isolate CTCs; therefore, the plasma (obtained after centrifugation at 800 *g*) containing the majority of EVs is discarded by default. As a consequence, more than 95% of the total tdEV population holding relevant clinical information is discarded. Nonetheless, the subset of isolated large EpCAM^+^, CK^+^ tdEVs from blood of cancer patients has a similar prognostic power to CTCs in metastatic prostate, breast, colorectal, and nonsmall cell lung cancer patients (Figure 12) and can complement CTCs in the CellSearch assay. Processing the plasma samples with the CellSearch assay and imaging of the enriched sample using a fluorescence microscope with a higher NA objective is expected to lead to increased tdEV detection with a higher offset when compared to the tdEV counts detected in the respective processed plasma samples of healthy individuals.

## Cancer-ID Insights

Cancer-ID delivered new techniques and new insights to explore tdEV detection. Taken the complexity of blood into consideration, the necessity of enriching biological samples for tdEVs becomes obvious. EVs secreted from prostate cancer cell lines and EVs derived from red blood cells and platelets, resembling the expected background of EVs in plasma, were used to explore the utility of different techniques. The size distribution of EV samples was characterized by NTA; the EV size and/or morphology by TEM, SEM, and AFM; their biochemical composition by Raman spectroscopy; and their antigen expression profile by SPRi, FM, and FCM. The techniques were able to detect or image EVs present in culture supernatants from tumor cells. However, discrimination between EVs and non-EV particles becomes difficult in complex samples like plasma because non-EV particles outnumber EVs (Figure 1). Furthermore, most techniques cannot identify the cellular origin of single EVs and relate the measured signal or count to the concentration of tdEVs in plasma. The results of all individual techniques pointed out that a combination of more than one parameters or techniques will increase the certainty that tdEVs are being investigated, and immune affinity enrichment or detection is needed to cover the large size and density range of EVs.

EV isolation protocols have not been standardized within the EV field (72, 73). Size-based isolation techniques, such as size exclusion chromatography, can purify samples from contaminating lipoproteins and soluble protein of a size below 70 nm (29). Furthermore, centrifugation is often used to isolate biomarkers from whole blood. In the Cancer-ID program, we developed a model to predict the behavior of particles (cells and EVs) in solution during centrifugation and showed the coisolation of, for example, platelets and large EVs after centrifugation (73). Moreover, although the application of rate zonal centrifugation improved the separation of platelets from EVs, the aforementioned isolation techniques result in purification of EVs rather than enrichment of tdEVs. Similarly, other techniques such as the asymmetric-flow field-flow fractionation can accurately separate exosomes and exomeres based on their size (74); however, for the characterization of EVs of interest, an additional pre-enrichment step will be required.

By the use of affinity-based techniques using antibodies directed to antigens expressed on tumor cells but not on blood cells, we demonstrated the enrichment of large (>1 μm) EpCAM^+^ tdEVs from blood from metastatic cancer patients (8, 9). EVs from different origin were eliminated in the enriched sample. Efforts for the immunomagnetic enrichment of smaller (<1 μm) tdEVs from plasma samples based on EpCAM are ongoing. The frequency of small tdEV shown in Figure 1 is based on an extrapolation from the frequency of the large tdEVs, and this surely will need to be validated. Moreover, whether the small tdEV have a similar relation with clinical outcome will need to be established. tdEV likely encompass different subclasses; for example, those responsible for communication with the environment and those involved in the process of apoptosis of cancer cells and as such relation with clinical outcome or its cargo being informative on the optimal treatment will likely be different between these subclasses. Here, only the EpCAM antigen was used to capture tdEVs; the use of different or a mixture of antibodies recognizing different cancer-specific antigens, such as VAR2CSA (75) and HsP70 (76, 77) could increase the capture efficacy and may identify different subclasses of tdEVs. Identification of tdEV among the EpCAM-enriched particles was obtained through identification of the presence of intracellular cytokeratins; the use of different components of the tdEV cargo might be important. Exploration of this cargo with label-free technologies such as Raman and SPRi identified some alternative avenues that can be explored. The onset of retrieving data from the molecular content of EVs has also been explored in the Cancer-ID program. A challenge is retrieving sufficient RNA to represent the messenger RNA (mRNA) and long noncoding RNA transcriptome. As a first step, various EV RNA isolation kits were tested, and of the isolation kits tested, the Norgen total RNA isolation protocol resulted in the highest amount of RNA as determined by reverse transcription quantitative PCR (RT-qPCR) of housekeeping and prostate-associated transcripts. Although this Norgen protocol will also extract non-EV RNA from urine, RNA yield and coverage by RNAseq are considered of higher priority than purity for our EV-based biomarker efforts.

State-of-the-art integrated systems developed in the Cancer ID Perspectief program come close to reliably detecting tdEVs at clinically relevant concentrations at high throughput. Small tdEVs (<1 μm) can be isolated using functionalized anti-EpCAM substrates and can be detected electrochemically in a label-free manner (19). Next, sorting of tdEV populations (as defined by fluorescence, by SPRi, electrochemically, or by Raman spectroscopy) can be used to perform downstream molecular analysis and reveal their genetic content that could play a critical role in identifying the best therapeutic strategy for cancer patients.

## Data Availability Statement

The datasets generated for this study are available on request to the corresponding author.

## Author Contributions

LR, PB, AE-M, AN, SL, CO, and LT drafted the manuscript. All other authors reviewed and improved on the draft.

## Conflict of Interest

The authors declare that the research was conducted in the absence of any commercial or financial relationships that could be construed as a potential conflict of interest. The handling Editor declared a past co-authorship with one of the authors LT.
